# Notes on Computational Uncertainties in Probabilistic Risk/Safety Assessment

**DOI:** 10.3390/e20030162

**Published:** 2018-03-04

**Authors:** Antoine Rauzy

**Affiliations:** Department of Mechanical and Production Engineering, Norwegian University of Science and Technology, 7491 Trondheim, Norway; Antoine.Rauzy@ntnu.no; Tel.: +47-918-97-151

**Keywords:** probabilistic risk/safety assessment, uncertainties, assessment algorithms, modeling methodologies

## Abstract

In this article, we study computational uncertainties in probabilistic risk/safety assessment resulting from the computational complexity of calculations of risk indicators. We argue that the risk analyst faces the fundamental epistemic and aleatory uncertainties of risk assessment with a bounded calculation capacity, and that this bounded capacity over-determines both the design of models and the decisions that can be made from models. We sketch a taxonomy of modelling technologies and recall the main computational complexity results. Then, based on a review of state of the art assessment algorithms for fault trees and event trees, we make some methodological proposals aiming at drawing conceptual and practical consequences of bounded calculability.

## 1. Introduction

A long journey has been made since the WASH 1400 report [1]. Probabilistic risk assessment (PRA) and probabilistic safety assessment (PSA) are nowadays widely accepted and deployed methods to assess risk of industrial systems such as nuclear power plants, offshore platform or aircrafts. Very large models combining fault trees and event trees are routinely used to make decisions about plant design and operations. Powerful tools are available to author and to assess these models.

This does not mean however that the PRA/PSA technology is mature and fully satisfactory. The famous quote by the statistician George Pellam Box “all models are false, some are useful” [2] applies indeed to PRA/PSA models. This statement should be constantly borne in mind when discussing the treatment of uncertainties in these models, which is the topics of the present article. More exactly, the different sources of “falsity” of models should be clearly understood and thoroughly weighted. It is actually questionable to perform long and complex mathematical developments to deal with uncertainties on some particular aspect of the modeling methodology if this aspect is at the end of the day like a drop in the bucket.

In this article, we explain why uncertainties coming from modeling formalisms and assessment algorithms take a very important place in the whole model uncertainty picture. Our experience is that this issue is often underestimated by both scientists and practitioners. This article aims thus at discussing the whys and wherefores of the current situation.

The key point here is that the calculation of probabilistic risk indicators is provably computational hard, namely #P hard, as demonstrated by Valiant [3] and further completed by Toda [4]. In practice, this means that PRA/PSA models result necessarily of a trade-off between the accuracy of the description of the system under study and the ability to perform calculations on this description. In other words, the risk analyst faces the fundamental epistemic and aleatory uncertainties of risk assessment with a bounded calculation capacity, and this bounded capacity over-determines both the design of models and the decisions that can be made from models. With that respect, he or she is like Simon’s economical agent who must make decisions with a bounded rationality [5].

The problem at stake can be thus formulated as follows: given my limited modeling and calculation capacities, and given all the uncertainties of the modeling process, where should I concentrate my efforts to ensure a reasonably correct and reasonably robust decision process?

This article is a contribution to answer this vast question. It gives the point of view of a computer scientist. It aims at drawing, from an engineering viewpoint, some consequences of bounded calculability.

The remainder of this article is organized as follows. Section 2 presents a high level view on the PRA/PSA modeling process and tries to locate the different sources of uncertainties. Section 3 establishes a taxonomy of PRA/PSA modeling formalisms and reviews fundamental computational complexity results regarding the calculation of risk indicators for the three categories of models defined by the taxonomy. Section 4 reviews state of the art algorithms for PRA/PSA Boolean model assessment and explains what makes them efficient in practice. Section 5 reports and discusses experimental results on large nuclear PSA models. Finally, Section 6 concludes the article.

## 2. The PRA/PSA Modeling Process

**Figure 1:**
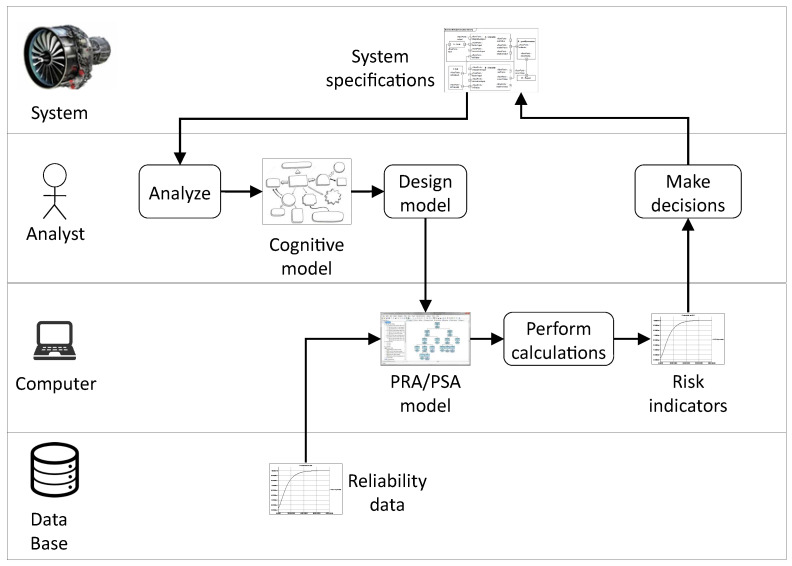
Idealized view of the PRA/PSA process.

Figure 1 shows an idealized view of the PRA/PSA process. It is worth following it step by step to discuss sources of uncertainties in models.

The first step of this process consists for the risk analyst in trying to understand how the system works and how it may fail. Functional analysis, as defined in reference textbooks [6,7], is typically part of this step although it does not cover it fully. The risk analyst works usually from system specifications and not from the system itself as the latter (or the configuation under study of the latter) may not exist yet.

One of the fundamental characteristics of risk/safety assessment is that it is usually not possible to adjust models by means of experiments on the system. Not only the latter may not exist at the moment of the analysis, but also the result of the analysis—roughly speaking the likelihood that something bad happens—is not directly measurable.

The first step is a large source of modeling uncertainties for several reasons including:

- The physical phenomena at stake may be only partially known and understood.
- The analyst may not master the mathematics (the physics, the chemistry, etc.) of these phenomena.
- System specifications may be incorrect or incomplete.
- The analyst may misunderstand these specifications for they are ambiguous.
- Some initiating events and their consequences within the system may escape the analyst’s attention.
- Interrelations between different system components and qualitative predictions of the time behavior in case of the occurrence of initiating events may be mistaken.

These uncertainties are usually called epistemic. This categorization is fine, but one should not forget that risk analyses are performed by individuals with their own knowledge and skills in an industrial process with its own technological and economical constraints. In other words, there may be a significant difference between the body of knowledge that could be relevant for the modeling process and the knowledge the analyst has and is able to use in practice.

The second step of the process consists in designing the actual PRA/PSA model. It reifies (from the Latin: to make thing) the cognitive model into a computerized one. This step takes also reliability data for basic components as input. These reliability data are stored into data bases such as OREDA [8]. The design of the PRA/PSA model is also a large source of uncertainties that must be examined thoroughly.

To design a computerized model, one needs a modeling language, just as to design a computer program one needs a programming language. As of today, most PRA/PSA models are designed using combinatorial modeling formalisms: fault trees, event trees, block diagrams or a combination of those. These formalisms make a strong assumption—the statistical independence of basic events— and for this reason have strong limitations: impossibility to represent faithfully cold redundancies, time dependencies, resource sharing, reconfigurations, etc. Combinatorial models are thus coarse approximations of behaviors of systems under study. Nevertheless, the use of these formalisms is decided a priori in most of PRA/PSA. Safety standards recommend them. To convince regulation bodies that alternative formalisms could be used is at best a long, a very long process. Consequently, risk analysts tend to reason in terms of combinatorial formalisms, even during the first step of the PRA/PSA process. This is fully understandable, for practical efficiency reasons, but this is also problematic in the sense that this keeps implicit and sometimes even undocumented the knowledge about approximations.

The fault tree/event tree/reliability block diagram methodology requires associating a probability distribution $U_{BE}\left(t\right)$ with each basic event BE of the model. Basic events represent failure modes of components of the system. $U_{BE}\left(t\right)$ characterizes thus the probability that the component is unavailable at time *t* in reason of the failure mode described by the basic event BE. In industrial practice, most of these probability distributions are either point estimates or parametric distributions—mainly exponential distributions and from time to time Weibull distributions. The parameters of these distributions are obtained from experience feedback on fleets of similar components used in similar conditions. Several important remarks can be made at this point:

- Probability distributions associated with basic events concentrate the aleatory uncertainty about behaviors of systems under study.
- Even if a large experience feedback has been accumulated over the years, the scarcity of reliable data is still an issue. The choice of parametric distributions such as the exponential distribution—which assumes a constant failure rate of the component over its mission time—is often made by default and for the sake of the conveniency rather than supported by strong empirical evidences, see, e.g., the introduction of the already cited OREDA handbook [8] for a discussion.
- Some margins can be taken to deal with the epistemic uncertainty about the aleatory uncertainty by considering probability distributions on parameters of probability distributions associated with basic events. The so-called sensitivity analyses— implemented in tools such as RiskSpectrum [9] and XFTA [10]—deal with these second order distributions.

We shall come back to these questions in details in the forthcoming sections.

The third step of the PRA/PSA process consists in calculating risk indicators from the model. Risk indicators include top event probability, importance factors, safety integrity levels and the like (see again reference textbooks [6,7] for a presentation). In most of the commercially available tools, these indicators are calculated from the minimal cutsets. More exactly, approximations of these indicators are calculated from the minimal cutsets. When probabilities of basic events are low and the model is not too large, these approximations are usually very good. When either of these two conditions is missing, results should be handled with care as we shall see in Section 4.

In any case, calculations of risk indicators are computationally expensive. Moreover, the richer is the modeling formalism, the more expensive is the calculations. This explains why formalisms more expressive than combinatorial formalisms are still seldom used in industrial practice.

In fact, the computational cost of risk indicators has a strong influence back on the whole PRA/PSA process: it determines the choice of modeling formalisms and through this choice the way analysts reason about the system.

The last step of the PRA/PSA process consists in making decisions about the system. These decisions are eventually quite simple: either the risk indicators show that the system is safe and reliable enough to be operated, or some changes have to be made (and the whole PRA/PSA cycle performed again).

In summary, PRA/PSA models have two main roles: first, their design helps risk analysts to review systems, and second, they are means to calculate risk indicators from which decisions can be made. They have several characteristics that make them quite different from models designed in other engineering disciplines:

- They are coarse approximations of the behavior of the system under study.
- It is nearly impossible to adjust them by means of experiments on the real system.
- Their assessment is computationally hard (in a sense we shall make precise in the next section), which over-determines their design and beyond their design, the way analysts reason about the system under study.

Nevertheless, they are the main, if not the only, tool at hand to assess the risk in complex technical systems. In other words, we have to live with epistemic, aleatory and computational uncertainties of risk assessment.

The scientific and technological challenge regarding PRA/PSA is thus to reduce these uncertainties as much as possible, given that modeling and calculation means are necessarily limited. With that respect, a key issue is to ensure that the decision process is reasonably robust, i.e., that small variations in models do not impact these decisions significantly. We shall study how to achieve this objective as efficiently as possible in the forthcoming sections.

## 3. The Computational Complexity Barrier

In this section, we review some important results about the computational complexity of assessment of PRA/PSA models.

### 3.1. Taxonomy of Modeling Formalisms

PRA/PSA models are made of two parts:

- A structural part describes how the system under study may fail under the occurrence of events such as failures, human errors, repairs, reconfigurations, etc.
- A probabilistic part associates probability distributions to the above mentioned events.

The structural part is independent of the probabilistic part.

PRA/PSA modeling formalisms can be divided roughly into three classes according to the expressive power of their structural part: (probabilized) Boolean formulas, (stochastic) finite state automata and (stochastic) process algebras. We shall consider them in turn.

#### 3.1.1. Probabilized Boolean Formulas

Probabilized Boolean formulas include fault trees, event trees, reliability block diagrams (see e.g., [6,7] for reference textbooks) and related formalisms such as Go-Flows [11], Dynamic Flow Graphs [12], multistate systems [13,14], and HiP-HOPS [15].

In these formalisms, the system under study is assumed to consist of a finite number *n* of components. Each component can be in a finite number of states, usually two (a component is either working or failed). The state the *i*th component, 1≤i≤n, is described by means of a variable $v_{i}$ that takes its value into a finite set of constants, like {0,1} where 0 stands for working and 1 stands for failed, called the domain of $v_{i}$ and denoted by $dom(v_{i})$. The state of the system is thus described by a vector $\bar{v}=\langle v_{1},\ldots ,v_{n}\rangle$ of variables that takes its value into the cartesian product $\prod_{i=1}^{n}dom\left(v_{i}\right)$ of the domains of the variables (which is indeed finite).

The set of states in which the system is failed is described by means of a Boolean formula $f(\bar{v})$ that is interpreted as a subset of $\prod_{i=1}^{n}dom\left(v_{i}\right)$.

Each variable $v_{i}$, 1≤i≤n, is equipped with a probability distribution, i.e., a function that associates with each value $c\in dom(v_{i})$ and each time *t* a certain probability $p_{v_{i}=c}\left(t\right)$.

It is assumed that components are statistically independent. Therefore, the probability that the system is in state $\bar{s}=\langle s_{1},\ldots ,s_{n}\rangle$ at time *t* is simply as follows.

(1) $$\begin{array}{ccc} p_{\bar{v}=\bar{s}}\left(t\right) & = & \prod_{i=1}^{n}p_{v_{i}=s_{i}}\left(t\right) \end{array}$$

From the above definitions, the following equality holds.

(2) $$\begin{array}{ccc} p_{f(\bar{s})}\left(t\right) & = & \sum_{\bar{s}\in f\left(\bar{v}\right)}p_{\bar{v}=\bar{s}}\left(t\right) \end{array}$$

In theory, $p_{f(\bar{v})}\left(t\right)$ is thus easy to assess. In practice, it is impossible to enumerate one by one all of the (failed) states of the system because of the exponential blow-up of their number (more on that point in the next section).

As already pointed out, probabilized Boolean formulas, because they assume components are statistically independent, have strong limitations: impossibility to represent faithfully cold redundancies, time dependencies, repairs, resource sharing, reconfigurations, etc.

#### 3.1.2. Stochastic Finite State Automata

Stochastic finite state automata include a large class of modeling formalisms such as Markov chains, (finite) stochastic Petri nets [16], (finite) guarded transition systems [17], dynamic fault trees [18], Boolean driven Markov processes [19], stochastic automata networks [20], stochastic extensions of Harel’s StateCharts (see, e.g., [21]) SAML [22], process algebras like PEPA [23] and PEPA-nets [24]…High level modeling languages such as Figaro [25] and AltaRica (in its successive versions: AltaRica LaBRI [26,27], AltaRica Data-Flow [28,29] and AltaRica 3.0 [30,31]) are other and more structured ways to describe finite state automata.

In these formalisms, the state of the system is still described by a vector $\bar{v}=\langle v_{1},\ldots ,v_{n}\rangle$ of variables that take their values into finite domains $dom(v_{i})$, 1≤i≤n. The set of states in which the system is failed is also still described by means of a Boolean formula $f(\bar{v})$ that is interpreted as a subset of $\prod_{i=1}^{n}dom\left(v_{i}\right)$.

The difference with probabilized Boolean formulas stands in the addition of:

- an initial state $\bar{\iota }$; and
- a finite set of transitions that describe how the system changes of state under the occurrence of events.

Transitions are triples 〈E,g,a〉, denoted $g\overset{E}{\to }a$, where:

- *E* is the event labeling the transition.
- *g* is a Boolean formula on the variables of $\bar{v}$. *g* is called the guard of the transition.
- *a* is an instruction that calculates the next values of the variables. *a* is called the action of the transition.

A transition $g\overset{E}{\to }a$ is fireable in a global state $\bar{s}$ if $g(\bar{s})=true$. Its firing transforms the state $\bar{s}$ into the state $a(\bar{s})$.

Except for Markov chains, the state space of the system is thus described implicitly: a given state $\bar{t}$ is reachable from the initial state $\bar{\iota }$ if either $\bar{t}=\bar{\iota }$, or there is another state $\bar{s}$ and a transition $T:g\overset{E}{\to }a$, such that $\bar{s}$ is reachable from $\bar{\iota }$, *T* is fireable in $\bar{s}$ and $a\left(\bar{s}\right)=\bar{t}$.

Each event *E* is equipped with a deterministic or probabilistic delay. The probability to be in the state $\bar{s}$ at time *t* is thus the sum of the probabilities of all possible sequences of transition firings that lead from state $\bar{\iota }$ at time 0 to state $\bar{s}$ at time *t*.

Stochastic finite state automata have indeed a much higher expressive power than probabilized Boolean formulas. They make it possible to represent faithfully cold redundancies, time dependencies, repairs, resource sharing, reconfigurations, etc. However, they describe finite state spaces and assume that its architecture does not change throughout its mission.

Note that several above mentioned formalisms entering into the class of stochastic finite state automata make it possible to describe infinite state spaces (e.g., Petri nets). Models are however designed in such way that the state space they describe stays finite.

#### 3.1.3. Stochastic Process Algebras

The last class of formalisms, stochastic process algebras, includes formalisms as diverse as (stochastic variants of) colored Petri nets (with an unbound number of colors) [32], process algebras such as Milner’s π-calculus [33], and agent-oriented modeling languages (see, e.g., [34] for an introduction). So-called “Systems of Systems” (see, e.g., [35] for a seminal article) can often be described in this way. Many calculation/simulation models or programming languages have been proposed in the literature that work more or less in this way (Simula has been historically the first one, see, e.g., [36]).

In these formalisms, the state of the system is also described as a vector $\bar{v}$ of variables encoding the individual states of its components and by transitions describing change of states, but:

- Some of the components may be in infinite number of different states (the domains of the corresponding variables are infinite).
- The size of the vector $\bar{v}$ may vary, as new components may be created and some existing components may be destroyed as the result of actions of transitions. The number of transitions may vary as well.

We gave here a presentation of models in terms of automata for the sake of uniformity. It is sometimes easier to see models of this class as descriptions of hierarchical processes running in parallel. Each component of the system is then seen as a process or an agent. During its execution, which may end before the end of the execution of the system as a whole, a process may “fork”, i.e., create some sub-processes or clone processes.

Formalisms belonging to this class have the full power of programming languages.

The three classes we mentioned in this section are ordered by increasing computational complexity of assessment algorithms, as we shall see now.

### 3.2. Computational Complexity

Computational complexity theory is a branch of theoretical computer science that aims at classifying problems according to the cost, in terms of computational resources, of solving them. We shall recall here only fundamental results related to PRA/PSA. The reader interested in a broader perspective should look at reference textbooks [37,38].

Computational complexity theory considers families of problems stated in mathematical terms. Of course, the cost of solving a problem must be related to the size of this problem. This size of problem can be measured for instance as the number of symbols required to encode this problem. It can be shown that, under reasonable assumptions, this is a suitable measure. The size of an instance *P* of a problem is denoted |P|.

The complexity of a problem is by definition the complexity of the best algorithm to solve that problem. The algorithm should indeed be able to solve any instance of the problem. The complexity of an algorithm is measured in terms of the number of steps this algorithm takes to solve the considered instance of the problem. As this number of steps may vary from one instance to the other, even if we consider instances of the same size, the complexity is characterized by means of a function f(n) such that for any instance of size *n* of the problem, the number of steps of the algorithm is at most c.f(n), for some predefined constant *c*. It is then said that this algorithm is in O(f(n)) (the big-O notation). For instance, sorting the element of a list using the quick-sort algorithm is in O(n.logn), where *n* denotes the number of elements of that list.

At this point, three important remarks can be made.

First, one can consider, aside this complexity in terms of the number of steps—called complexity in time—the complexity in terms of number of memory cells required by the algorithm—called complexity in space. Complexity in time provides in general a better understanding of the actual cost of calculations, but we shall see that the complexity in space is usefull as well.

Second, we are speaking here of worst-case complexity. It is also possible to consider average complexity, but results are then much more difficult to establish.

Third, we assumed in the above discussion that the problems at stake are decidable, i.e., that there exist at least one algorithm to solve them. Some important practical problems (for instance the equivalence of two computer programs) are however undecidable, i.e., it can be proven that no general algorithm exists to solve them.

Decidable problems fall in one of the three following categories, with respect to their complexity.

- Provably easy problems, i.e., those for which algorithms with polynomial complexity are known. These problems are said to be P-easy. Some of them are also P-hard, meaning that no algorithm with a lower complexity than polynomial can be designed to solve them.
- Provably hard problems, i.e., those for which it can be proved that any algorithm has at least an exponential complexity. These problems are said to be EXP-hard.
- Problems that are neither provably easy nor provably hard. There is a wide variety of very practical such problems.

The above classification is rather rough as a problem in $O(n^{100})$ can hardly be considered as easy in any practical sense. However, very few such problems have been exhibited so far, so the classification is widely accepted.

Until now, we spoke about problems in general. We need now to be more specific and to distinguish decision problems and enumeration problems. A decision problem is a problem with an answer that is either yes or no. An enumeration problem is a problem that consists in counting the number of yes answers of a decision problem or, if a probability structure is defined over the possible answers, in assessing the sum of the probabilities of the yes answers.

Here, another two important remarks can be made.

First, a common point to decision and enumeration problems is that their answer can be encoded in a small space compared to the size of the problem. This is not the case for all of the problems. For instance, the encoding of the set of reachable states of a finite state automaton may be exponentially larger than the encoding of the automaton itself (not to speak about the set of reachable states of a process algebra model that can be infinite while the description of the automaton itself is finite).

Second, enumeration problems are indeed at least as hard, and in general much harder, than their decision counterpart. If we know how to count the number of solutions to a problem, we know a fortiori if there is a solution to this problem.

### 3.3. Complexity of PRA/PSA Assessment

#### 3.3.1. The Six Central Problems of PRA/PSA Assessment

We can now come to the complexity of PRA/PSA assessment. The key risk/safety indicator is indeed the probability that the system is in a failed state at time *t*. The complexity of calculating this probability depends obviously of the class of the model at stake. To characterize this complexity, it is necessary to study also the complexity of the corresponding decision problem. We can thus formulate the following six central problems of PRA/PSA assessment.

SAT: Let $f(\bar{v})$ be a Boolean formula built over a set of variables $\bar{v}$. Is there a valuation $\bar{s}$ of $\bar{v}$ such that $f(\bar{s})=true$?

RELIABILITY: Let $f(\bar{v})$ be a Boolean formula built over a set of variables $\bar{v}$. Assume moreover that $\bar{v}$ is equipped with a probability structure (as defined above). What is the probability of *f* (i.e., the sum of probabilities of variable valuations $\bar{s}$ such that $f(\bar{s})=true$)?

REACHABILITY: Let *M* be a finite state automaton. Is there a reachable failed state, i.e., is there a sequence of transitions starting from the initial state of *M* and leading to a failed state?

FSA-RELIABILITY: Let *M* be a finite state automaton equipped with a probability structure (as defined above). What is the probability to reach a failed state at time *t*?

PA-REACHABILITY: Let *M* be a process algebra model. Is there a reachable failed state, i.e., is there a sequence of transitions starting from the initial state of *M* and leading to a failed state?

PA-RELIABILITY: Let *M* be a process algebra model equipped with a probability structure (as defined above). What is the probability to reach a failed state at time *t*?

SAT, RELIABILITY and REACHABILITY are “official” names [37,38]. We defined the others for the purpose of the present article.

We shall now review known computational complexity results about the above problems.

#### 3.3.2. Complexity PRA/PSA Assessment based on Probabilized Boolean Formulas

SAT plays a central role in computational complexity theory.

A first remark is that it is easy to check whether a candidate variable valuation $\bar{s}$ satisfies *f* by propagating bottom-up values of variables in the formula. The algorithm to do so is of linear worst case complexity with respect to the size of the formula. The problem is indeed that there are potentially $2^{n}$ valuations to check if *f* involves *n* variables (and each variable can take two values).

The class NP is the class of decision problems having the same characteristic as SAT, i.e., such that, given a candidate solution, it is easy to check whether it is actually a solution but there are exponentially many candidate solutions. NP stands for non-deterministic polynomial. Obviously, P⊆NP⊂EXP.

In 1971, Cook demonstrated the following theorem.

**Theorem** **1** (Complexity of SAT[39]). SAT is NP-complete, i.e., any problem of the class NP is reducible to SAT, i.e., can be transformed in polynomial time into a SAT instance that has a solution if and only if the problem has one.

The following question is one of the most intriguing of computer science.

$$\begin{array}{ccc} P & \overset{?}{=} & \mathrm{NP} \end{array}$$

As of today, it is still open.

Note that MONOTONE-SAT, i.e., the variant of SAT in which the formula *f* is coherent (monotone), is trivially an easy problem (according to the our classification): it suffices to check whether the valuation that assigns the value true to all variables satisfies *f* because if *f* is satisfied by a valuation it must be satisfied by that one as well. We shall give in the next section a formal definition of coherence.

The class #P (read “sharp P”, or “number P”) gathers counting and reliability problems associated with NP-hard problem (i.e., problems that are at least as hard as problems in the class NP). For instance, #SAT is defined as follows.

#SAT: Let *f* be a Boolean formula. How many variable valuations satisfy *f*?

This class has been introduced by Valiant [3] who showed the following theorem.

**Theorem** **2** (Complexity of #SAT [3]). #SAT is #P-complete.

The two following additional properties are easy to show (see [38]).

**Property** **1** (RELIABILITY versus #SAT). RELIABILITY is at least as hard as #SAT.

**Property** **2** (#MONOTONE-SAT versus #SAT). #MONOTONE-SAT, i.e., the problem of counting the number of solutions of a coherent formula, is as hard as #SAT.

Valiant’s theorem has been later completed by Toda.

**Theorem** **3** (Toda[4]). PP is as Hard as the Polynomial-Time Hierarchy

It would go far beyond the scope of this paper to explain Toda’s theorem. Intuitively, it says that if one can count “for free” the number of solutions of a problem, then one is able to solve in polynomial time all of the problems of the polynomial hierarchy, i.e., is very close to be able to solve in polynomial time problems of exponential worst case complexity.

In a word, RELIABILITY is strongly believed to be a hard problem. We shall elaborate further on this topics Section 4 and explain why, despite of these negative results, very large fault trees and related models can be efficiently assessed, thanks to the coherence of models and to suitable approximations.

#### 3.3.3. Complexity PRA/PSA Assessment based on Stochastic Finite State Automata

The following theorem establishes the complexity of REACHABILITY.

**Theorem** **4** (Complexity of REACHABILITY [38]). REACHABILITY is PSPACE-complete.

The above theorem asserts that REACHABILITY can be solved in polynomial space and that any problem in this class can be reduced to REACHABILITY.

The good news is that, despite the fact that there may be a exponential number of reachable states, one can decide in polynomial space whether a failed state is reachable. This result is obtained by accepting to redo some calculations, i.e., pass several times by the same state. The bad news is that the above result is not very useful in practice and that it cannot anyway be applied to the calculation of the probability of being in a failed state at time *t*. The following theorem formalizes this negative result.

**Theorem** **5** (Complexity of FSA-RELIABILITY). FSA-RELIABILITY is EXP-hard.

The key remark here is that the number of states on sequences leading to failed states may be exponentially large with respect to the size of the problem. FSA-RELIABILITY is thus a hard problem, with all respects.

As of today, two approaches have been proposed to solve FSA-reliability in practice: the compilation of the model into a Markov chain and stochastic simulation.

A first approach consists thus in compiling, when possible, the model into a Markov chain, and then to apply numerical algorithms to solve Markov chains, see, e.g., [40] for a reference book on these numerical methods and [41] for a study dedicated to reliability models. This approach suffers indeed from the exponential blow-up of the number of states and transitions of the Markov chain. It is however possible to compute approximated Markov chains, with good practical results, see, e.g., [42].

The second approach consists performing Monte-Carlo simulations. Monte-Carlo simulation is the Swiss knife of models engineering in general and reliability engineering in particular, see, e.g., [43] for a recent monograph. It is feasible if the probability to be calculated is not too low (the number of runs required to get reasonably accurate results increases with the inverse of this probability).

In summary, stochastic finite state automata are a reasonable alternative to probabilized Boolean formulas when the system at stake presents characteristics that cannot be faithfully captured by a pure combinatorial model. Assessing stochastic finite state automata is however extremely intensive in terms of calculation resources even if only reasonably good approximations of the values of risk indicators are required. As of today, the use of stochastic finite state automata is thus limited to relatively small models with relatively high values of risk indicators.

#### 3.3.4. Complexity PRA/PSA Assessment based on Stochastic Process Algebras

As the reader may expect, the situation gets even worse for process algebra models. Namely, almost any relevant question on these models is undecidable.

**Theorem** **6** (Complexity of PA-REACHABILITY). PA-REACHABILITY is undecidable.

The above result follows directly from results on severe restrictions of this general problem. For instance, the reachability problem applied to Petri nets with inhibitor arcs is already undecidable [44].

An immediate consequence of the above theorem is that PA-RELIABILITY is also undecidable.

These undecidability results explain probably why process algebras are seldom used for practical reliability studies. The gain in terms of expressive power over stochastic finite state automata is obtained at a too high price.

Note that it is nevertheless still possible to apply to this class of models the approaches developed for stochastic finite state automata, namely the compilation into approximated Markov chains (when possible) and more importantly, stochastic simulation. The author is convinced that this class of models will play an increasingly important role in the future. The key issue however stands in the validation of such models.

### 3.4. Wrap-Up

In this section, we proposed a taxonomy of modeling formalisms that can be used to support PRA/PSA analyses. We review known computational complexity results. They are essentially bad news: assessing risk indicators is an intractable problem, except for the very specific case where the model is coherent fault tree (or an equivalent representation).

This explains why, despite the strong limitations of this class of models, they are almost exclusively used in PRA/PSA practical applications.

We shall study them in further details in the next section.

Before proceeding, we would like to emphasize here that the computational complexity of PRA/PSA assessment is one of the contributors to epistemic uncertainty. It comes in addition to other contributors such as those mentioned in Section 2. The problems raised by computational complexity stand in the impossibility to model the system faithfully because of the complexity of assessments.

## 4. Assessment Algorithms for Probabilized Boolean Formulas

In this section, we review state of the art assessment algorithms for probabilized Boolean formulas. Understanding how these algorithms work is actually mandatory to handle uncertainties in a proper way. Nevertheless, we shall not enter into technical details, but rather present the principles. In depth presentations can be found in articles by the author [45,46].

### 4.1. Taxonomy of Assessment Algorithms

PRA/PSA models like fault trees, event trees, reliability block diagrams and the like are eventually interpreted as Boolean formulas built over the two constants 0 (false) and 1 (true), a finite set of variables, so-called basic events, and logical connectives “+” (or), “·” (and) and “(¯” (not). Other connectives such as *k*-out-of-*n* can be easily derived from those.

The calculation of all risk indicators is based on a basic step consisting in calculating the probability of a Boolean formula *f*, given the probabilities of basic events of *f*, which is nothing but the RELIABILITY problem stated in the previous section.

As *f* may contain repeated events, it is not possible in general to calculate p(f) directly from *f*. *f* must transformed into an equivalent normalized formula from which the calculation is possible. Two normal forms have been proposed so far: sums-of-minimal-cutsets and binary decision diagrams.

**Figure 2:**
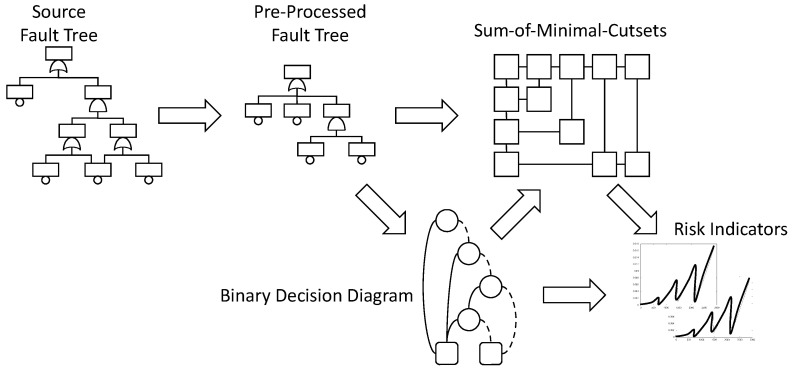
The PRA/PSA calculation flow.

Figure 2 summarizes the calculation flow.

Starting from the initial fault tree (or from the master fault tree generated from a fault tree/event tree model), one pre-processes the model to make it easier to assess. This first step involves notably the detection of modules, i.e., of sub-formulas that are independent from the rest of the model and can thus be assessed separately. The importance of module detection has been pointed out since the very first work on fault tree assessment [47] and is still an essential ingredient of it. Efficient algorithms have been proposed detect modules (e.g., [48]), so the preprocessing phase, although extremely important regarding the overall assessment efficiency, is not itself very resource consuming.

Once the model preprocessed, the hard things start. There is here an alternative with the two above mentioned branches: either a sum-of-minimal-cutsets, or a binary decision diagram is calculated. Minimal cutsets represent failure scenarios. They are of interest on their own, even if no quantification takes place. That is the reason why algorithms have been designed to calculate minimal cutsets from binary decision diagrams [45].

The last step of the assessment consists in calculating risk indicators, either from the sum-of-minimal-cutsets or from the binary decision diagram, depending on which normal form has been chosen. Risk indicators include the top event probability, possibly for different mission times, importance factors, safety integrity levels and some others. Efficient algorithms exist to calculate these indicators, see, e.g., Reference [49] for importance factors and Reference [50] for safety integrity levels.

As sum-of-minimal-cutsets and binary decision diagrams play a central role in the whole assessment process, we shall now give more insights about what they are and how they are calculated.

### 4.2. Minimal Cutsets

A literal is either a basic event or its negation. A product is a conjunction of literals that does not contain both a basic event and its negation. A product is positive if it contains no negated basic event. A sum of products is a set of products interpreted as their disjunction. Two products are disjoint if there is at least one basic event occurring positively in one of them and negatively in the other. A sum of disjoint products (SDP) is a sum of products whose products are pair wisely disjoint. A minterm relatively to a set of basic events is a product that contains a literal for each basic event in the set. By construction, two different minterms are disjoint. Minterms one-to-one correspond with truth assignments of basic events (we called them system states in the previous section). For that reason, the following property holds.

**Property** **3** (Sum-of-Minterms). Any Boolean formula is equivalent to a unique sum of minterms.

Let *f* and *g* be two formulas built over the same set of basic events. We denote by $Minterms\left(f\right)$ the sum of minterms equivalent to the formula *f*. We say that the minterm π satisfies the formula *f*, and denote π∈f, if π belongs to Minterms(f) and that it falsifies *f* otherwise. Similarly, we write f⊆g, if $Minterms\left(f\right)\subseteq Minterms\left(g\right)$, i.e., if *f* entails *g*, and f≡g if Minterms(f)=Minterms(g), i.e., if *f* and *g* are logically equivalent. Note that logical equivalence is the strongest possible equivalence relation over models. Two logically equivalent models are indistinguishable by any correct quantification algorithm.

Let π and ρ be two minterms. We say that π is smaller than ρ, which we denote as π≤ρ, if any basic event that occurs positively in π occurs positively in ρ.

A Boolean formula *f* is coherent if for any two minterms π and ρ such that π≤ρ, π∈f implies ρ∈f. It is easy to verify that any formula built only over basic events and connectives “+” and “.” is coherent.

Let π be a positive product. We denote by ⌊π⌋ the minterm built by completing π with the negative literals built over basic events that do not show up in π. In other words, ⌊π⌋ is the smallest minterm ρ such that ρ∈π.

A cutset of a Boolean formula *f* is defined as a positive product π, such that ⌊π⌋∈f. A cutset π is minimal if no sub-product of π is a cutset of *f*. We shall denote by $\mathrm{MCS}\left(f\right)$ the set (the sum) of minimal cutsets of the formula *f*. The following property holds [45].

**Property** **4** (Minimal Cutsets). *Let f be a Boolean formula. Then, $f\subseteq MCS\left(f\right)$. Moreover:*

- $f\equiv MCS\left(f\right)$ if and only if f is coherent.
- $\mathrm{MCS}\left(f\right)$ is the smallest coherent formula containing f, i.e., $\mathrm{MCS}\left(f\right)\subseteq g$ for any coherent formula g such that f⊆g.

One way of understanding Property 4 is to say that coherent systems are perfectly represented by their minimal cutsets but that for non-coherent systems minimal cutsets are a (sometimes very) conservative approximation of the original model.

Two categories of algorithms have been proposed to calculate minimal cutsets directly from a (pre-processed) fault tree:

- Top-down algorithms, which are derived from MOCUS [51]. Such algorithms are implemented in Risk Spectrum [9] and XFTA [10,52].
- Bottom-up algorithms, which use Minato’s zero-suppressed binary decision diagrams [53] to encode minimal cutsets. Such an algorithm is implemented in FTREX [54], one of the calculation engines of CAFTA.

In theory, the calculation of the probability of a sum-of-minimal-cutsets can be performed thanks to the Sylvester-Poincaré development.

**Property** **5** (Sylvester-Poincaré development). *Let $f=\sum_{i=1}^{n}\pi_{i}$ be a sum-of-products. Then, the following equality holds.*

$$\begin{array}{cc} p(f)= & \sum_{1\leq i\leq n}p\left(\pi_{i}\right)-\sum_{1\leq i_{1}<i_{2}\leq n}p\left(\pi_{i_{1}}\;\cdot \;\pi_{i_{2}}\right)\\ & +\ldots +-1^{k-1}\sum_{1\leq i_{1}<\ldots <i_{k}\leq n}p\left(\pi_{i_{1}}\;\cdot \;\ldots \;\cdot \;\pi_{i_{k}}\right)+\ldots +-1^{n-1}p\left(\pi_{1}\;\cdot \;\ldots \;\cdot \;\pi_{n}\right) \end{array}$$

where the probability of a product is the product of the probabilities of its literals.

In practice however, the computational cost of this calculation method is prohibitive as it involves the calculation of $2^{n}$ terms, where *n* is the number of minimal cutsets. Approximations are thus performed:

- The so-called rare-event approximation that consists in considering only the first term of the development.
  $$\begin{array}{ccc} p_{REA}\left(f\right) & \overset{def}{=} & \sum_{\pi \in \mathrm{MCS}\left(f\right)}p\left(\pi \right) \end{array}$$
- The so-called mincut upper bound approximation, which warranties, conversely to the rare-event approximation, to get a result between 0 and 1.
  $$\begin{array}{ccc} p_{MCUB}\left(f\right) & \overset{def}{=} & 1-\prod_{\pi \in \mathrm{MCS}\left(f\right)}\left(1-p\left(\pi \right)\right) \end{array}$$

Both approximations are accurate when the probabilities of basic events are small (say less than $10^{-2}$).

### 4.3. Binary Decision Diagrams

Binary decision diagrams are a data structure making it possible to encode in a very compact way the truth table of (many) Boolean functions and to perform operations (conjunction, disjunction, negation, etc.) on these functions. They have been introduced in their modern form by R. Bryant and his colleagues [55,56].

Binary decision diagrams rely on the pivotal or Shannon decomposition.

**Property** **6** (Pivotal decomposition). *Let f be a Boolean formula and E be a basic event (occurring in f). Then the following equivalence holds.*

$$\begin{array}{ccc} f & \equiv & E\;\cdot \;f_{E=1}+\bar{E}\;\cdot \;f_{E=0} \end{array}$$

where $f_{E=v}$ denotes the formula f in which the constant v has been substituted for the basic event E.

Technically, binary decision diagrams are directed acyclic graphs with two types of nodes:

- Leaves 〈c〉 that are labeled with a Boolean constant c∈{0,1}. Leaves are interpreted as the constant they are labeled with:
  $$\begin{array}{ccc} ⟦\langle c\rangle ⟧ & \overset{def}{=} & c \end{array}$$
- Internal nodes 〈E,v,w〉 that are labeled with a basic event *E* and have two out-edges: a then out-edge pointing to the node *v*, and an else out-edge pointing to the node *w*. Binary decision diagrams are constructed in such a way that the basic event *E* never shows up in the sub-trees rooted by nodes *v* and *w*. Internal nodes are interpreted as pivotal decompositions:
  $$\begin{array}{ccc} ⟦\langle E,v,w\rangle ⟧ & \overset{def}{=} & E\;\cdot \;⟦v⟧+\bar{E}\;\cdot \;⟦w⟧ \end{array}$$

Binary decision diagrams encode thus formulas fully decomposed according to Property 6. They are built bottom-up: the binary decision diagram encoding a formula is obtained by applying Boolean operations on binary decision diagrams encoding its sub-formulas.

Binary decision diagrams have been introduced in the reliability field at the beginning of the nineties [57]. They have proven since then to outperform all other assessment methods—when it is possible to build the binary decision diagram encoding the top event of the fault tree under study. It is not always the case when dealing with large models (with several hundred basic events and more) as the binary decision diagram may be too large to fit into the computer memory (and even on external hard disks).

One of the key features of binary decision diagrams is that they make the calculation of the top event probability both exact (no approximation is required) and of linear complexity, thanks to the following property (that results from Property 6).

**Property** **7** (Pivotal decomposition applied to probabilities). *Let f be a Boolean formula and E be a basic event (occurring in f). Then the following equivalence holds.*

$$\begin{array}{ccc} p(f) & = & p\left(E\right)\times p\left(f_{E=1}\right)+\left(1-p\left(E\right)\right)\times p\left(f_{E=0}\right) \end{array}$$

To compute the exact probability of the function represented by means of a binary decision diagram it suffices thus to calculate recursively the probability of each node of the diagram. This principle applies also for the calculation of conditional probabilities and Birnbaum importance factor [49].

### 4.4. Consequences of Computational Complexity Results

Let us summarize the situation by putting together computational complexity results reviewed in the previous section and the algorithms presented above:

1. Fault trees can be assessed in two ways:
  - Either by preprocessing the tree, extracting its minimal cutsets and then approximating the top event probability from the minimal cutsets;
  - Or by preprocessing the tree, building its binary decision diagram and then calculating the exact top-event probability.
2. RELIABILITY is (strongly believed to be) a hard problem.
3. Preprocessing the tree, approximating the top event probability from the minimal cutsets, and calculating the exact top event probability from the binary decision diagram are easy operations.

This implies that:

- Either extracting minimal cutsets is a hard problem, or obtaining a good approximation of the top event probability from minimal cutsets is a hard problem, or both.
- Building the binary decision diagram is a hard problem.

These theoretical results are confirmed in practice: the three above operations are actually intractable, at least if we take them in their whole generality.

### 4.5. Approximations

At this point, the reader may think: “All right, this is for the problem in general, but in practice, given the epistemic uncertainties on the system behavior, on its modeling and on reliability data, I’m just fine with reasonable approximations.” and she or he is right to think so. The question is: what does mean “reasonable” here?

If no constraint is put on the model, finding accurate approximations seems in fact almost as hard as finding the exact value as demonstrated by several partial results by Ball and Provan, see e.g., [58,59,60].

However, Boolean PRA/PSA models have two essential characteristics.

First, they are coherent. Even when formulas embeds some negations, these negations are used as a shortcut to represent exclusive configurations and not to reflect a “real” non coherence, see [46] for a discussion. This the reason why they can be assessed by means of minimal cutsets algorithms (which are always coherent). This is not surprising as one can expect that the more components are failed in a mechanical system, the more likely this system is failed. We shall come back on this issue in the next section.

Second, they represent highly reliable systems made of highly reliable components. This translates into the following inequality for most, if not all, of the basic events of the model.

(3) $$\begin{array}{ccc} p_{E}\left(t\right) & \ll & p_{\bar{E}}\left(t\right) \end{array}$$

It follows that large minimal cutsets and minterms with a high number of positive literals have a very low probability and can be safely ignored. In other words, one can focus on failure scenarios involving few faulty components because scenarios involving many faulty components are highly improbable.

These two characteristics are combined into state of the art algorithms to calculate accurate approximations of risk indicators. It works as follows.

First, a probabilistic weight is defined on products as follows. Let π be a product.

$$\begin{array}{ccc} w(\pi ) & \overset{def}{=} & \prod_{E\in \pi }p\left(E\right) \end{array}$$

That is, w(π) is the product of the probabilities of positive literals of π.

Now, given a formula *f* and a probability threshold τ, we can define the following restrictions of *f* and $\mathrm{MCS}\left(f\right)$ with τ as follows.

$$\begin{array}{ccc} f_{\geq \tau } & \overset{def}{=} & \sum_{\pi \in Minterms\left(f\right);\;w\left(\pi \right)\geq \tau }\pi \\ {\mathrm{MCS}}_{\geq \tau }\left(f\right) & \overset{def}{=} & \left\{\pi \in \mathrm{MCS}\left(f\right);\;w\left(\pi \right)\geq \tau \right\} \end{array}$$

The following property holds.

**Property** **8** (Minimal Cutsets of Restrictions [45]). *Let f be a Boolean formula and τ be a probability threshold, then:*

$$\begin{array}{ccc} MCS\left(f_{\geq \tau }\right) & = & MCS_{\geq \tau }\left(f\right) \end{array}$$

Moreover, under the condition that most of the basic events verify the inequality 3, the probability of *f* at time *t* can be accurately approximated as follows (via the calculation of ${\mathrm{MCS}}_{\geq \tau }\left(f\right)$).

(4) $$\begin{array}{ccc} p(f) & \approx & p_{REA}\left(f_{\geq \tau }\right) \end{array}$$

(5) $$\begin{array}{ccc} p(f) & \approx & p_{MCUB}\left(f_{\geq \tau }\right) \end{array}$$

Let $p_{lb}$ be the probability of the basic event with lowest probability. Clearly, any product π with *k* positive literals verifies $w\left(\pi \right)\geq p_{lb}^{k}$. Therefore, if the cutoff τ is chosen such that $\tau \leq p_{lb}^{k}$, only minimal cutsets (and minterms) with most *k* positive literals need to be considered when calculating $p_{REA}\left(f_{\geq \tau }\right)$ (or $p_{MCUB}\left(f_{\geq \tau }\right)$). However, there is only a polynomial number of such products, since there is a polynomial number to select at most *k* items in a set of *n* items.

It follows that $p_{REA}\left(f_{\geq \tau }\right)$ (and $p_{MCUB}\left(f_{\geq \tau }\right)$) are polynomial approximations of p(f). They can be calculated via the two alternative algorithmic approaches described above: either by extracting only the minimal cutsets whose probability is higher than τ or by calculating an approximated binary decision diagram, cutting branches encoding a product π such that w(π)<τ, see [45] for more details. In both cases, it is possible to track what has been discarded so to get an upper bound of the actual probability.

This very positive result, which makes PRA/PSA of practical interest, should not hide the epistemic problems it raises, due to the following paradox.

Assume we designed a model *M* at a given level of details. We calculated from *M* minimal cutsets and relevant risk indicators with a probability threshold τ. As we did our job as correctly as possible, we set up τ as low as possible for the available calculation power.

Now, assume that for some reason, we decide to refine the model *M* into a model $M^{\prime }$. $M^{\prime }$ decomposes certain basic events into gates so to analyze with a finer grain the failure modes of some components. A priori, results obtained from *M* and $M^{\prime }$ should be equivalent. The difference stands in the fact that a minimal cutset of *M* can be refined into a group of minimal cutsets of $M^{\prime }$.

However, here come two problems. First, as $M^{\prime }$ is larger than *M* and generates thus possibly many more minimal cutsets than *M*, the probability threshold τ may be too small for $M^{\prime }$ and the available calculation power. We are thus forced to make the calculations with a coarser probability threshold $\tau^{\prime }$ ($\tau^{\prime }>\tau$). Second, a minimal cutset π of *M* whose probability was above to the threshold τ, may be decomposed into several minimal cutsets whose probabilities are below τ and therefore below $\tau^{\prime }$. It follows that these minimal cutsets will be discarded while assessing $M^{\prime }$.

We are thus in the following paradoxical situation.

**Paradox** **1** (Model refinement). The more refined is the model, the lower is the risk estimation.

By refining sufficiently the model, we could even make the (evaluated) risk vanish completely.

### 4.6. Handling Uncertainties on Reliability Data

Probability distributions of basic events of PRA/PSA models are known only up to an uncertainty. This problem has many causes, including the scarcity of data, that have been discussed at length in the abundant literature on this topics. We shall not attempt to review these contributions here, as they are not at the core of our subject, but just have a look at how uncertainties are handled in practice when calculating risk indicators.

To simplify the discussion, we shall assume that the mission time of the system is fixed and that probabilities of basic events are calculated at this mission time. Saying that the probability p(E) of the basic event *E* is known only up to an uncertainty, is saying that it belongs to a certain interval $\left[p_{min}\left(E\right),p_{max}\left(E\right)\right]$. The density of probability in this interval has no reason to be uniformly distributed. It can be for instance normally distributed (taking into account truncations due to bounds) arround a certain value.

Assuming given such interval (and density probability within the interval) for each basic event of the model/formula *f*, we can attempt to characterize the uncertainty in the calculation of p(f).

The range of variation of the probability of the formula can be significantly larger than the individual range of variations of the probabilities of the basic events. To understand this phenomena, consider a minimal cutset $\pi =E_{1}\;\cdot \;\ldots \;\cdot \;E_{k}$. Then, $p_{min}\left(\pi \right)=\prod_{i=1}^{k}p_{min}\left(E_{i}\right)$ and $p_{max}\left(\pi \right)=\prod_{i=1}^{k}p_{max}\left(E_{i}\right)$. Consequently, if $p_{min}\left(E_{i}\right)=\rho_{i}\times p_{max}\left(E_{i}\right)$ for i=1,…,k, then $p_{min}\left(\pi \right)=\prod_{i=1}^{k}\rho_{i}\times p_{max}\left(\pi \right)$. The same reasoning applies to each minimal cutset and therefore for $p_{min}\left(f\right)$ and $p_{max}\left(f\right)$. In other words, individual uncertainties multiply.

For this reason, just performing interval calculation gives in general much too coarse results on industrial size models. Two main alternative methods have been proposed: first, to use extended probability theories, such as the Dempster–Shafer theory [61]; and, second, to perform Monte-Carlo simulations on probabilities of basic events. Both methods have their own advantages and drawbacks.

Extended probability theories make it possible to perform calculations efficiently. However, they do not really solve the above problem. Moreover, determining the degree of belief or plausibility of the failure of a component from field data is a quite difficult task.

With that respect, the Monte-Carlo simulation approach seems more practical. However, it is extremely consuming in terms calculation resources. This is the reason why, simulations are usually performed on the same set of minimal cutsets (or the same binary decision diagram), obtained for a probability threshold τ and the mean values of basic event probabilities. It would be actually too costly to recompute the minimal cutsets (or the binary decision diagram) for each set of probabilities of basic events.

The next section presents experimental results on industrial use cases that illustrate the different points discussed above.

## 5. Experimental Results

To illustrate the different points discussed in the previous section, we selected three large models out of our benchmarks. These three models comes from the nuclear industry. These models are extracted from PSA studies of an American and two European nuclear power plants (from two different European countries).

The numbers of basic events and gates of these models are as follows.

**PSA Model****#Basic Events****#Gates**117331304223125346328165583

Each of these models represents a group of sequences of an event tree model leading to a nuclear accident (e.g., core melt). Models 1 and 2 are non coherent in the sense that they embed negated gates and basic events to represent exclusive or impossible configurations, see, e.g., [46] for a discussion on this issue.

We assessed these models with XFTA [10,52], the fault tree calculation engine the author develops in the framework of the Open-PSA initiative [62,63]. XFTA is a very efficient fault tree calculation engine. It is free of use under unrestrictive conditions.

Experiments reported here have been performed on a PC under Windows 10, with a Intel(R) Core(TM) 64 bits processor cadenced at 2.40 GHz with 8 GB memory. This PC has been bought at the local supermarket.

### 5.1. Calculation of Minimal Cutsets and the Top-Event Probability

Table 1, Table 2 and Table 3 report the results obtained on, respectively, Models 1, 2 and 3. They are organized as follows.

Each row of the table corresponds to a different cutoff value. We took as cutoffs the negative powers of 10, ranging from the first value for which at least one minimal cutset is produced to a value where the top event probability is stabilized.

Note that the critical resource here is the memory rather than the computation time. Thanks to XFTA data structures, it is possible to store about 60 millions minimal cutsets within our computer memory. Beyond, the tool has to be configured specifically, which we did not want to do (we wanted results to be reproducible with the distributed version of XFTA).

The columns of the tables report the following information.

- The first column gives the value of the cutoff.
- The second and third columns give the top event probability computed from the minimal cutsets with respectively the rare event approximation and the mincut upper bound.
- The fourth column gives the number of minimal cutsets.
- The fifth column gives the number of different basic events showing up in the minimal cutsets.
- The sixth column gives, in percentage, the ratio of the value of the rare event approximation obtained for the given threshold and the value of the rare event approximation obtained with the lower cutoff we could calculate with (i.e., the value indicated in the second cell of the last row of the table).
- The seventh column gives, in percentage, the ratio of the number of basic events showing up in the minimal cutsets over the total number of basic events of the model.
- Finally, the eighth column gives the running time in seconds for the whole calculation.

We can already draw several important conclusions from this first series of experiments.

First, XFTA is very efficient. It makes it possible to assess very large models, with millions of minimal cutsets, within seconds where other tools take minutes, if not hours, and are not able to compute with cutoffs as low as reported here. At a first glance, this may seem in contradiction with the development we made throughout this article. However, it is not, or not fully. On the one hand, XFTA results of decades of intensive research on algorithm and heuristics. On the other hand, models under study are nearly coherent Boolean formulas for which polynomial time approximations exist, as explained in the previous section. We shall discuss this issue in more details later in the section.

Second, there is not much difference between the results provided by rare event approximation and those obtained with the mincut upper bound. This is due to the fact that minimal cutsets have low probabilities. The benefit of using the latter approximation is thus limited (especially if we balance it with its algorithmic cost).

Third, in the three models, very few minimal cutsets and thus very few basic events, concentrate the most part of the accident probability. Moreover, even when calculating with a very low cutoff value, a significant part of the basic events does not show up in the minimal cutsets. In other words, there is a significant difference between the model as designed and the model as calculated. This calls for the development of tools that would synthesize the calculated model from the designed model and the list of basic events showing up in the minimal cutsets. This means also that the efforts to reduce uncertainties should probably be focused on these few important minimal cutsets and their basic events.

Fourth, the number of minimal cutsets grows steadily as the cutoff decreases. The minimal cutsets with a low probability do not contribute much to the top event probability. However, they have a strong impact on other risk measures like importance measures. Importance measures such as the Birnbaum importance factor, the Risk Achievement Worth and the Risk Reduction Worth, which are extensively used in nuclear PSA studies, discard the probability of the basic event they are measuring, see [49] for a detailed discussion about this topics. Some authors criticized them for this very reason, see, e.g., [64]. However, the key point here is that the ranking of basic events may show a chaotic behavior with respect to the selected cutoff value. This phenomenon has been first pointed out in Reference [65] and confirmed on a larger extent by Duflot et al. [66,67].

### 5.2. Testing the Robustness of the Results

Testing the robustness of the results is indeed of primary importance when assessing the safety of a critical system. This applies especially to the robustness of the assessment of the top event probability, given the existing uncertainties on reliability data, i.e., on probabilities of basic events (or parameters of probability distributions from which these probabilities are obtained).

As pointed out in the previous section, there are several methods to do so, including interval calculations, interpretation of probabilities into an extended logic (such as the Dempster–Shafer theory), and Monte-Carlo simulation.

As we are seeking here for general results, we shall adopt a slightly different approach. The idea is to study the impact of a variation in the same direction of the probability of all basic events. This method is probably a good way to test the robustness of the results obtained with nominal probabilities of basic events.

A first test consists in making probabilities of basic events vary slightly. Table 4, Table 5 and Table 6 report results obtained by increasing by 10% the probabilities of basic events of the three models.

These tables are organized as previously. The only difference stands in the sixth column: the reference probability, i.e., the denominator of the ratio, is the one of the previous table so to make clear the difference on the top event probability induced by the slight increase of basic event probabilities.

The probability of the top event is not very impacted by this slight change in basic event probabilities. The increases are respectively of 30%, 60% and 40%.

The numbers of minimal cutsets for each value of the cutoff vary in a similar way. There is an increase, but this increase is not too drastic.

Note that the increase in the top event probability is mostly due to the increase in basic event probabilities and not to the increase in the number of minimal cutsets, at least for the smallest values of the threshold.

The picture changes radically when we consider a more significant change of basic events probabilities. Table 7, Table 8 and Table 9 report results obtained by multiplying by 2 the probabilities of basic events of the three models. Note that such a variation, although very significant, is not unrealistic given the epistemic uncertainties on these probabilities.

Now top event probabilities are respectively multiplied by 10, 26 and 17! The number of minimal cutsets is also very significantly bigger for each value of the cutoff. However, as previously, the increase in the top event probability is mostly due to the increase in basic event probabilities and not to the increase in the number of minimal cutsets.

Some calculations that were possible become intractable. In any case, running times are significantly increased.

Note that the same observation applies the other way round as well: if we divide by a factor 2 the probabilities of the basic events, we divide by a factor much greater than 2 the probability of the top event.

Roughly speaking, if we consider, for each basic event *E* of “mean” probability $p_{E}$ the range $\left[p_{E}/\rho ,p_{E}\times \rho \right]$, for a certain factor ρ≥1, then the top event probability will vary in the interval $\left[p_{top}/\rho^{k},p_{top}\times \rho^{k}\right]$, where $p_{top}$ is the probability calculated for the mean values of basic event probabilities and *k* is the “mean” length of minimal cutsets.

This second series of experiments bring good news and bad news. The good news is that is may not be necessary to recompute the minimal cutsets in each run of a Monte-Carlo simulation (on basic event probabilities). Just recomputing the top event probability from the minimal cutsets calculated with the mean values of basic event probabilities is probably sufficient. The bad news is that, if the uncertainties on basic event probabilities are not small, the uncertainty in the top event probability may be so large that this central indicator looses it significance. In this case, other methods (than Monte-Carlo simulation or interval calculation) have to be put in place. A good idea is probably to perform a case study on the probability of the most important basic events. This is fairly possible because, as we have shown, there are not so many such basic events.

### 5.3. Discussion

The results given in this section are puzzling and lead to the following paradox.

**Paradox** **2** (Feasibility of calculations). Although involving the resolution of theoretically intractable problems, state of the art cutoff based algorithms make it possible to assess very large PRA/PSA models.

We could take this paradox just as another illustration of the famous quote: “In theory there is no difference between theory and practice. In practice there is”. However, this is indeed rather unsatisfying, especially because it is easy to exhibit trivial formulas for which the algorithms do not give any good results: For any value of the cutoff τ, consider the disjunction of *n* similar basic events whose probability *p* is lower than τ. Clearly, a cutoff based algorithm detects none of the singleton cutsets and therefore estimates the probability of the formula to 0. However, by letting *n* growing, we can make the probability of the formula arbitrarily close to 1, i.e., the error of the algorithm as big as we want.

This calls for a characterization of the formulas for which cutoff based algorithms work. This could work as follows.

Let *f* be a formula built over a set of variables V and let τ be a cutoff value. We can split $Minterms\left(f\right)$ into two subsets:

- The set $Minterms_{\geq \tau }\left(f\right)$ of minterms whose probabilistic weight is greater or equal to τ.
- The set $Minterms_{<\tau }\left(f\right)$ of minterms whose probabilistic weight is less than τ.

The absolute error $\sigma_{\tau }\left(f\right)$ and the relative error $\rho_{\tau }\left(f\right)$ on the estimation of the probability of *f* made by a cutoff based algorithm for a given value of τ can be characterized as follows.

$$\begin{array}{ccc} \sigma_{\tau }\left(f\right) & \overset{def}{=} & p\left(Minterms_{<\tau }\left(f\right)\right)\\ \rho_{\tau }\left(f\right) & \overset{def}{=} & \frac{p\left(Minterms_{<\tau }\left(f\right)\right)}{p\left(Minterms\left(f\right)\right)} \end{array}$$

These measures can be used in two ways: for a given value of the cutoff τ, they characterize the relative and absolute errors made by a cutoff based algorithm, and for a given value ϵ of the relative or absolute error we are ready to accept, they characterize the value of the cutoff to be used.

This characterization of probabilized Boolean formulas is quite different from other complexity measures proposed in the literature. The Shannon’s entropy, as introduced by Shannon in [68], can be used to characterize the amount of information in minterms and therefore in formulas. Intuitively, the elements of $Minterms_{\geq \tau }\left(f\right)$ tend to have a low Shannon’s entropy while those of $Minterms_{<\tau }\left(f\right)$ tend to have a high Shannon’s entropy. The problem is indeed that the Shannon’s entropy of *f* considers both $Minterms_{\geq \tau }\left(f\right)$ and $Minterms_{<\tau }\left(f\right)$, i.e., does not make approximations. For the same reason, trying to characterize approximatable formulas by the size of their normal form (which can be seen as a kind of Kolmogorov complexity) or the computational cost of obtaining it (which can be seen as a kind of Benett’s logical depth) is not satisfying, see, e.g., [69] for a reference book on these notions. Eventually, the closest notion one can find is probably the probably approximately correct learning (PAC learning) introduced by Valiant in [70] to ground the computational learning theory, see also [71]. Here, the set of hypotheses would be the set of all possible normal forms for formulas whose minterms have probabilistic weight lower than the cutoff τ and the concept to be learned would be the normal form for $Minterms_{\geq \tau }\left(f\right)$.

## 6. Conclusions

In this article, we studied the uncertainties in probabilistic risk/safety assessment (PRA/PSA) due to the computational complexity of assessment of risk indicators.

First, we proposed a taxonomy of modeling formalisms used in the PRA/PSA context. We reviewed known complexity results for these formalisms and showed that, except for the very particular case where the support model is a nearly coherent probabilized Boolean formula (i.e., can be translated into a nearly coherent fault trees), calculations at stake are intractable. This comes in some sense as an a posterio theoretical justification of a well established practice. We argued that this is also contributing to a large extent to the epistemic uncertainty on systems under study because this latter class of models does not allow to represent faithfully however important features of systems involving dependencies amongst events.

In a second step, we reviewed state of the art assessment algorithms for the assessment of nearly coherent fault trees and related models. We showed that these algorithms actually calculate polynomial approximations of risk indicators, and, provided the probabilities of basic events are low enough, these approximations are accurate. This good news comes however with an epistemic price that we called the model refinement paradox: the more detailed the model, the lower the risk estimation.

Finally, we reported the results of an experimental study on three large PSA models coming from the nuclear industry. This study showed that in these models at least: (i) a few minimal cutsets (and thus basic events) concentrate the probability of the top event; (ii) the number of extracted minimal cutsets grows steadily with the decrease of the cutoff; and (iii) even for low values of the cutoff, a large proportion of basic events do not show up in the extracted minimal cutsets. This has at least two important consequences in terms of epistemic uncertainty: first, there is a real discrepancy between the model as designed and the model as assessed; and, second, risk indicators such as importance measures may show a chaotic behavior with respect to the selected cutoff. We illustrated finally that, although results are quite robust to small variations of basic event probabilities, the uncertainties on the latter’s accumulate. Consequently, even not too large uncertainties on basic event probabilities may produce a large uncertainty on risk indicators.

The above theoretical and experimental results should not be taken as a criticism of the probabilistic approach in reliability engineering. Just the contrary: By better understanding its advantages and possible drawbacks, we delimit better its scope and make it a powerful and trustable tool. With that respect, much remains to do in terms of mathematical, algorithmic and experimental developments, to take a better benefit of this approach.

Assessing the risk in critical systems is and will remain a complex task. The analyst has definitely to face aleatory and epistemic uncertainties, and to face them with a limited computation power. This echoes in the engineering domain Simon’s bounded rationality of economic agents. The question at stake is eventually how to be efficient in the modeling process given our bounded computation resources.

## Figures and Tables

**Table 1:** Results obtained on Model 1 (1733 basic events, 1304 gates).

Cutoff	REA	MCUB	#MCS	#BE	REA%	BE%	Time (s)
1.00$\times 10^{-5}$	3.55000$\times 10^{-4}$	3.54966$\times 10^{-4}$	3	4	74.50%	0.2%	0.07
1.00$\times 10^{-6}$	4.03857$\times 10^{-4}$	4.03805$\times 10^{-4}$	20	26	84.75%	1.5%	0.16
1.00$\times 10^{-7}$	4.28640$\times 10^{-4}$	4.28578$\times 10^{-4}$	122	108	89.95%	6.2%	0.42
1.00$\times 10^{-8}$	4.50211$\times 10^{-4}$	4.50139$\times 10^{-4}$	924	237	94.48%	13.7%	1.01
1.00$\times 10^{-9}$	4.65220$\times 10^{-4}$	4.65141$\times 10^{-4}$	6120	429	97.63%	24.8%	2.34
1.00$\times 10^{-10}$	4.71964$\times 10^{-4}$	4.71882$\times 10^{-4}$	29,098	755	99.04%	43.6%	5.39
1.00$\times 10^{-11}$	4.74889$\times 10^{-4}$	4.74805$\times 10^{-4}$	124,582	1055	99.66%	60.9%	12.62
1.00$\times 10^{-12}$	4.75985$\times 10^{-4}$	4.75901$\times 10^{-4}$	480,930	1166	99.89%	67.3%	27.59
1.00$\times 10^{-13}$	4.76365$\times 10^{-4}$	4.76281$\times 10^{-4}$	1,693,755	1323	99.97%	76.3%	61.00
1.00$\times 10^{-14}$	4.76491$\times 10^{-4}$	4.76407$\times 10^{-4}$	5,658,636	1464	99.99%	84.5%	137.00
1.00$\times 10^{-15}$	4.76529$\times 10^{-4}$	4.76445$\times 10^{-4}$	17,579,596	1515	100.00%	87.4%	288.00

**Table 2:** Results obtained on Model 2 (2312 basic events, 5346 gates).

Cutoff	REA	MCUB	#MCS	#BE	REA%	BE%	Time (s)
1.00$\times 10^{-7}$	6.48254$\times 10^{-7}$	6.48254$\times 10^{-7}$	4	11	11.41%	0.5%	0.18
1.00$\times 10^{-8}$	2.11285$\times 10^{-6}$	2.11284$\times 10^{-6}$	57	41	37.20%	1.8%	0.39
1.00$\times 10^{-9}$	3.40600$\times 10^{-6}$	3.40599$\times 10^{-6}$	590	149	59.96%	6.4%	0.89
1.00$\times 10^{-10}$	4.40506$\times 10^{-6}$	4.40505$\times 10^{-6}$	4222	348	77.55%	15.1%	2.20
1.00$\times 10^{-11}$	5.07637$\times 10^{-6}$	5.07636$\times 10^{-6}$	27,543	687	89.37%	29.7%	6.03
1.00$\times 10^{-12}$	5.42694$\times 10^{-6}$	5.42693$\times 10^{-6}$	146,831	1095	95.54%	47.4%	15.77
1.00$\times 10^{-13}$	5.58671$\times 10^{-6}$	5.58670$\times 10^{-6}$	682,050	1464	98.35%	63.3%	39.99
1.00$\times 10^{-14}$	5.65404$\times 10^{-6}$	5.65403$\times 10^{-6}$	2,908,473	1711	99.54%	74.0%	104.00
1.00$\times 10^{-15}$	5.68026$\times 10^{-6}$	5.68024$\times 10^{-6}$	11,459,524	1919	100.00%	83.0%	280.00

**Table 3:** Results obtained on Model 3 (2816 basic events, 5583 gates).

Cutoff	REA	MCUB	#MCS	#BE	REA%	BE%	Time (s)
1.00$\times 10^{-7}$	7.31207$\times 10^{-7}$	7.31207$\times 10^{-7}$	3	6	18.51%	0.2%	0.40
1.00$\times 10^{-8}$	2.00813$\times 10^{-6}$	2.00812$\times 10^{-6}$	55	76	50.84%	2.7%	0.76
1.00$\times 10^{-9}$	3.08379$\times 10^{-6}$	3.08379$\times 10^{-6}$	457	243	78.07%	8.6%	1.14
1.00$\times 10^{-10}$	3.62022$\times 10^{-6}$	3.62022$\times 10^{-6}$	2421	495	91.65%	17.6%	2.60
1.00$\times 10^{-11}$	3.83641$\times 10^{-6}$	3.83640$\times 10^{-6}$	10,005	912	97.13%	32.4%	4.11
1.00$\times 10^{-12}$	3.91496$\times 10^{-6}$	3.91495$\times 10^{-6}$	36,717	1301	99.12%	46.2%	7.56
1.00$\times 10^{-13}$	3.94020$\times 10^{-6}$	3.94019$\times 10^{-6}$	119,767	1577	99.75%	56.0%	14.29
1.00$\times 10^{-14}$	3.94738$\times 10^{-6}$	3.94737$\times 10^{-6}$	350,488	1797	99.94%	63.8%	27.97
1.00$\times 10^{-15}$	3.94930$\times 10^{-6}$	3.94929$\times 10^{-6}$	958,104	1955	99.98%	69.4%	52.00
1.00$\times 10^{-16}$	3.94979$\times 10^{-6}$	3.94977$\times 10^{-6}$	2,473,798	2084	100.00%	74.0%	98.00
1.00$\times 10^{-17}$	3.94990$\times 10^{-6}$	3.94984$\times 10^{-6}$	6,074,179	2179	100.00%	77.4%	182.00

**Table 4:** Results obtained by increasing by 10% the probabilities of basic events of Model 1.

Cutoff	REA	MCUB	#MCS	#BE	REA%	BE%	Time (s)
1.00$\times 10^{-5}$	4.41529$\times 10^{-4}$	4.41474$\times 10^{-4}$	4	6	92.66%	0.3%	0.08
1.00$\times 10^{-6}$	5.00144$\times 10^{-4}$	5.00062$\times 10^{-4}$	24	31	104.96%	1.8%	0.19
1.00$\times 10^{-7}$	5.39665$\times 10^{-4}$	5.39563$\times 10^{-4}$	170	118	113.25%	6.8%	0.48
1.00$\times 10^{-8}$	5.72057$\times 10^{-4}$	5.71937$\times 10^{-4}$	1339	265	120.05%	15.3%	1.15
1.00$\times 10^{-9}$	5.93737$\times 10^{-4}$	5.93604$\times 10^{-4}$	8579	473	124.60%	27.3%	2.73
1.00$\times 10^{-10}$	6.03324$\times 10^{-4}$	6.03185$\times 10^{-4}$	41,377	827	126.61%	47.7%	6.41
1.00$\times 10^{-11}$	6.07380$\times 10^{-4}$	6.07239$\times 10^{-4}$	173,891	1082	127.46%	62.4%	14.95
1.00$\times 10^{-12}$	6.08905$\times 10^{-4}$	6.08763$\times 10^{-4}$	667,433	1190	127.78%	68.7%	33.69
1.00$\times 10^{-13}$	6.09427$\times 10^{-4}$	6.09285$\times 10^{-4}$	2,345,094	1351	127.89%	78.0%	75.00
1.00$\times 10^{-14}$	6.09599$\times 10^{-4}$	6.09456$\times 10^{-4}$	7,707,230	1489	127.92%	85.9%	166.00
1.00$\times 10^{-15}$	6.09650$\times 10^{-4}$	6.09508$\times 10^{-4}$	23,883,995	1523	127.94%	87.9%	352.00

**Table 5:** Results obtained by increasing by 10% the probabilities of basic events of Model 2.

Cutoff	REA	MCUB	#MCS	#BE	REA%	BE%	Time (s)
1.00$\times 10^{-7}$	1.20217$\times 10^{-6}$	1.20217$\times 10^{-6}$	6	12	21.16%	0.5%	0.20
1.00$\times 10^{-8}$	3.45096$\times 10^{-6}$	3.45095$\times 10^{-6}$	80	48	60.75%	2.1%	0.45
1.00$\times 10^{-9}$	5.73216$\times 10^{-6}$	5.73214$\times 10^{-6}$	954	193	100.91%	8.3%	1.08
1.00$\times 10^{-10}$	7.32196$\times 10^{-6}$	7.32193$\times 10^{-6}$	7002	428	128.90%	18.5%	2.86
1.00$\times 10^{-11}$	8.33469$\times 10^{-6}$	8.33465$\times 10^{-6}$	42,736	766	146.73%	33.1%	7.94
1.00$\times 10^{-12}$	8.86488$\times 10^{-6}$	8.86484$\times 10^{-6}$	222,655	1172	156.06%	50.7%	20.51
1.00$\times 10^{-13}$	9.10702$\times 10^{-6}$	9.10699$\times 10^{-6}$	1,030,887	1529	160.33%	66.1%	52.81
1.00$\times 10^{-14}$	9.20761$\times 10^{-6}$	9.20757$\times 10^{-6}$	4,358,927	1775	162.10%	76.8%	140.00
1.00$\times 10^{-15}$	9.24656$\times 10^{-6}$	9.24652$\times 10^{-6}$	17,060,713	1946	162.78%	84.2%	390.00

**Table 6:** Results obtained by increasing by 10% the probabilities of basic events of Model 3.

Cutoff	REA	MCUB	#MCS	#BE	REA%	BE%	Time (s)
1.00$\times 10^{-7}$	1.10523$\times 10^{-6}$	1.10523$\times 10^{-6}$	4	11	27.98%	0.4%	0.44
1.00$\times 10^{-8}$	2.87116$\times 10^{-6}$	2.87115$\times 10^{-6}$	71	97	72.69%	3.4%	0.69
1.00$\times 10^{-9}$	4.26522$\times 10^{-6}$	4.26521$\times 10^{-6}$	560	269	107.98%	9.6%	1.28
1.00$\times 10^{-10}$	4.99193$\times 10^{-6}$	4.99191$\times 10^{-6}$	2982	522	126.38%	18.5%	2.39
1.00$\times 10^{-11}$	5.28019$\times 10^{-6}$	5.28017$\times 10^{-6}$	12,362	962	133.68%	34.2%	4.48
1.00$\times 10^{-12}$	5.38284$\times 10^{-6}$	5.38283$\times 10^{-6}$	45,166	1336	136.28%	47.4%	8.38
1.00$\times 10^{-13}$	5.41505$\times 10^{-6}$	5.41504$\times 10^{-6}$	145,340	1612	137.09%	57.2%	16.07
1.00$\times 10^{-14}$	5.42414$\times 10^{-6}$	5.42414$\times 10^{-6}$	423,962	1816	137.32%	64.5%	30.79
1.00$\times 10^{-15}$	5.42654$\times 10^{-6}$	5.42653$\times 10^{-6}$	1,156,010	1974	137.38%	70.1%	57.47
1.00$\times 10^{-16}$	5.42715$\times 10^{-6}$	5.42713$\times 10^{-6}$	2,987,579	2098	137.40%	74.5%	110.00
1.00$\times 10^{-17}$	5.42730$\times 10^{-6}$	5.42722$\times 10^{-6}$	7,320,431	2192	137.40%	77.8%	205.00

**Table 7:** Results obtained by multiplying by 2 the probabilities of basic events of Model 1.

Cutoff	REA	MCUB	#MCS	#BE	REA%	BE%	Time (s)
1.00$\times 10^{-5}$	2.56231$\times 10^{-3}$	2.55952$\times 10^{-3}$	49	43	537.70%	2.5%	0.18
1.00$\times 10^{-6}$	3.44500$\times 10^{-3}$	3.43956$\times 10^{-3}$	400	145	722.94%	8.4%	0.48
1.00$\times 10^{-7}$	4.20053$\times 10^{-3}$	4.19221$\times 10^{-3}$	3210	301	881.48%	17.4%	1.39
1.00$\times 10^{-8}$	4.66314$\times 10^{-3}$	4.65277$\times 10^{-3}$	19,527	586	978.56%	33.8%	3.41
1.00$\times 10^{-9}$	4.89122$\times 10^{-3}$	4.87977$\times 10^{-3}$	96,421	888	1026.43%	51.2%	8.51
1.00$\times 10^{-10}$	4.98920$\times 10^{-3}$	4.97727$\times 10^{-3}$	419,437	1138	1046.99%	65.7%	22.03
1.00$\times 10^{-11}$	5.02556$\times 10^{-3}$	5.01344$\times 10^{-3}$	1,603,024	1285	1054.62%	74.1%	52.51
1.00$\times 10^{-12}$	5.03848$\times 10^{-3}$	5.02629$\times 10^{-3}$	5,706,077	1438	1057.33%	83.0%	128.00
1.00$\times 10^{-13}$	5.04259$\times 10^{-3}$	5.03038$\times 10^{-3}$	18,723,478	1516	1058.19%	87.5%	291.00
1.00$\times 10^{-14}$	5.04383$\times 10^{-3}$	5.03162$\times 10^{-3}$	57,063,870	1553	1058.45%	89.6%	677.00

**Table 8:** Results obtained by multiplying by 2 the probabilities of basic events of Model 2.

Cutoff	REA	MCUB	#MCS	#BE	REA%	BE%	Time (s)
1.00$\times 10^{-7}$	4.09621$\times 10^{-5}$	4.09613$\times 10^{-5}$	113	46	721.13%	2.0%	0.47
1.00$\times 10^{-8}$	7.85630$\times 10^{-5}$	7.85599$\times 10^{-5}$	1529	187	1383.09%	8.1%	1.33
1.00$\times 10^{-9}$	1.07583$\times 10^{-4}$	1.07577$\times 10^{-4}$	12,561	485	1893.98%	21.0%	3.79
1.00$\times 10^{-10}$	1.27490$\times 10^{-4}$	1.27482$\times 10^{-4}$	84,354	931	2244.44%	40.3%	11.57
1.00$\times 10^{-11}$	1.38664$\times 10^{-4}$	1.38655$\times 10^{-4}$	471,371	1364	2441.16%	59.0%	33.16
1.00$\times 10^{-12}$	1.44230$\times 10^{-4}$	1.44220$\times 10^{-4}$	2,357,504	1674	2539.14%	72.4%	97.00
1.00$\times 10^{-13}$	1.46712$\times 10^{-4}$	1.46701$\times 10^{-4}$	10,620,675	1882	2582.84%	81.4%	296.00

**Table 9:** Results obtained by multiplying by 2 the probabilities of basic events of Model 3.

Cutoff	REA	MCUB	#MCS	#BE	REA%	BE%	Time (s)
1.00$\times 10^{-7}$	2.81395$\times 10^{-5}$	2.81391$\times 10^{-5}$	84	84	712.41%	3.0%	0.59
1.00$\times 10^{-8}$	4.90584$\times 10^{-5}$	4.90572$\times 10^{-5}$	840	232	1242.02%	8.2%	1.06
1.00$\times 10^{-9}$	5.96707$\times 10^{-5}$	5.96690$\times 10^{-5}$	4355	498	1510.69%	17.7%	2.13
1.00$\times 10^{-10}$	6.38861$\times 10^{-5}$	6.38841$\times 10^{-5}$	18,007	869	1617.41%	30.9%	4.18
1.00$\times 10^{-11}$	6.53132$\times 10^{-5}$	6.53111$\times 10^{-5}$	63,358	1295	1653.54%	46.0%	8.30
1.00$\times 10^{-12}$	6.57587$\times 10^{-5}$	6.57565$\times 10^{-5}$	202,389	1608	1664.82%	57.1%	16.96
1.00$\times 10^{-13}$	6.58864$\times 10^{-5}$	6.58842$\times 10^{-5}$	594,713	1818	1668.05%	64.6%	33.93
1.00$\times 10^{-14}$	6.59207$\times 10^{-5}$	6.59186$\times 10^{-5}$	1,644,065	1977	1668.92%	70.2%	67.00
1.00$\times 10^{-15}$	6.59296$\times 10^{-5}$	6.59274$\times 10^{-5}$	4,307,856	2108	1669.15%	74.9%	132.00
1.00$\times 10^{-16}$	6.59317$\times 10^{-5}$	6.59296$\times 10^{-5}$	10,727,093	2207	1669.20%	78.4%	256.00
1.00$\times 10^{-17}$	6.59322$\times 10^{-5}$	6.59299$\times 10^{-5}$	25,482,478	2271	1669.21%	80.6%	500.00
